# All-cause and cause-specific mortality in older people with and without diabetes in Norwegian home care services: a nationwide registry study

**DOI:** 10.1186/s12877-025-06685-z

**Published:** 2025-12-05

**Authors:** Tonje Teigland, Jannicke Igland, Kjersti M. Blytt, Johannes Haltbakk, Kåre I. Birkeland, Truls Østbye, Marit Kirkevold, Marjolein M. Iversen

**Affiliations:** 1 Department of Health and Caring Sciences, Western Norway University of Applied Sciences, Bergen, Norway; 2 Department of Global Public Health and Primary Care, University of Bergen, Bergen, Norway; 3 Institute of Clinical Medicine, University of Oslo, Oslo, Norway; 4 Department of Transplantation Medicine, Oslo University Hospital, Oslo, Norway; 5 Department of Family Medicine and Community Health, Duke University, Durham, NC USA; 6 Faculty of Health Sciences, Oslo Metropolitan University, Oslo, Norway

**Keywords:** Mortality, Diabetes, Home care services, Older people, Registry

## Abstract

**Background:**

As the number of older people living at home increases, the role of home care services (HCS) becomes more important. Diabetes is associated with premature mortality and is common among older people in HCS, yet mortality patterns among HCS recipients are not well known. This study aimed to estimate all-cause and cause-specific mortality risk in persons with pharmacologically treated diabetes receiving HCS compared to other HCS recipients and explore whether mortality risk differed between diabetes treatment subgroups.

**Methods:**

This nationwide registry study merged data from the Norwegian Information System for the Nursing and Care Sector with data from the Norwegian Prescription Database, the Norwegian Patient Registry, and the Cause of Death Registry (CDR). The study population included recipients of HCS (aged 65–90 years at baseline) in Norway between 2009 and 2014. Individuals were classified as having pharmacologically treated diabetes (hereafter referred to as diabetes) (≥ 1 prescription of glucose-lowering drugs (GLD) in the current half year or the year before), or not having diabetes. Those with diabetes were further sub-classified into “non-insulin GLD only”, “insulin and non-insulin GLD”, or “insulin only”. Time of death and the underlying cause of death were retrieved from CDR. The study population, diabetes status, covariates, and all-cause mortality were updated each half-year. Mortality risk was compared between groups using Cox proportional hazards regression, with age as time scale, and reported as hazard ratio (HR) with 95% CIs.

**Results:**

Women in the “insulin only” group had a higher risk of all-cause mortality (HR 1.18 (CI 1.11–1.25)) than women without diabetes, while in the “non-insulin GLD only” and “insulin and non-insulin GLD” subgroups, both women and men with diabetes had lower mortality risks than those without diabetes. Overall, persons with diabetes had a higher risk of cardiovascular mortality (HR 1.23 (CI 1.19–1.28)) compared to persons without diabetes, and a lower risk of dying from cancer (HR 0.68 (CI 0.66–0.70)) and respiratory disease (HR 0.67 (CI 0.62–0.72)).

**Conclusion:**

Mortality risk varied by diabetes status and treatment subgroups. Most diabetes subgroups had lower all-cause mortality risk than those without diabetes, except for women using “insulin only”, underscoring the need for individualized HCS.

**Supplementary Information:**

The online version contains supplementary material available at 10.1186/s12877-025-06685-z.

## Background

The growing life expectancy in combination with an epidemic of non-communicable diseases presents a significant challenge to health care systems worldwide [1, 2]. The demographic shift towards an aging population likely implies a prolonged period of living with poor health, frailty, and disability [3]. In high-income regions, almost 50% of the total disease burden is attributable to disorders in older adults (defined as aged 60 years or older) [1]. Addressing health priorities and age-appropriate care for chronic diseases are among the key challenges arising from this demographic trend [1]. Furthermore, the prevalence of diabetes is increasing, and by 2045, 20% (276 million) of individuals aged 65–99 years may have diabetes [3].

Diabetes is associated with premature mortality due to cardiovascular disease (CVD), cancer, and other non-cardiovascular non-cancer causes [4], although recent studies suggest declining trends in mortality and a shift in predominant causes of death among persons with diabetes [5, 6]. Gregg et al. found that among 46 million adults in England, individuals with diabetes who had three or more diabetes-associated long-term conditions by the age of 50 had a life expectancy 11 years shorter than the general population [7]. Others have found a decline in the excess vascular mortality among persons with diabetes, while cancer has been identified as the new leading contributor to the excess mortality observed among persons with diabetes compared to persons without diabetes [8]. Differences in mortality risk between women and men with diabetes have also been identified [9, 10]. Forbes et al. [11] found that the excess mortality in older people with diabetes compared to people without diabetes could not be explained by comorbidity. Still, the excess mortality among older people with diabetes is found to be 10% higher than in the general population, highlighting the need for further research and to identify optimal care strategies for this population [9].

With the increasing population of older people and extended responsibilities for community-based healthcare services, home care services (HCS) have become increasingly important [12–14]. A study using data from five European countries reported declines in both physical and cognitive skills among home care clients in the period from 2001 to 2014, underscoring their need for substantial support [14]. Although several recent studies have examined older individuals receiving HCS, epidemiological research in this area remains limited, and research specifically addressing mortality risk in this population is scarce. Frailty and multimorbidity are common in older age, and while frailty is associated with increased need for home care and risk of nursing home admissions, mortality is more strongly associated with the number of diseases [2]. It is still unclear whether the differences in mortality risk observed between people with and without diabetes in the general population also apply to older individuals receiving HCS. A clearer understanding of mortality patterns and associated risk factors in persons with and without diabetes receiving HCS may facilitate more targeted and effective interventions at the system, institutional, and individual levels.

Information on all-cause and cause-specific mortality risks can provide valuable epidemiological insights into older adults with diabetes in HCS, thereby enhancing our understanding of risk factors and informing potential interventions. Such knowledge may also help identify vulnerable groups within HCS who could benefit from a more individualized and targeted medical approach. Therefore, the primary aim of this study was to estimate all-cause and cause-specific mortality risk in persons with pharmacologically treated diabetes receiving HCS compared to other HCS recipients and explore whether all-cause and cause-specific mortality risk differed between diabetes treatment subgroups. Additionally, we described the distribution of the underlying causes of death in persons with and without pharmacologically treated diabetes, and across different treatment subgroups.

## Methods

### Study design and study population

In this nationwide registry study, data from the Norwegian Information System for the Nursing and Care Sector (IPLOS) for the study population were merged with data from the Norwegian Prescription Database (NorPD), Norwegian Patient Registry (NPR), Cause of Death Registry (CDR) and Statistics Norway (SSB). The study population consists of all persons aged 65–90 years receiving HCS in Norway between 2009 and 2014 (at least one hour or one day in a given half-year).

### Setting: home care services in Norway

In Norway, as in many other countries, the policy is to enable older people to remain in their homes for as long as possible, with health care delivered at the “lowest level of effective care” [15]. This approach seeks to optimize resource allocation while minimizing the strain on the healthcare system. Norway has extensive public regulations and funding of the healthcare system, and municipalities are responsible for allocating and delivering HCS to applicants [15]. Older people may apply for HCS for various reasons, such as physical and/or mental illness, impaired health, the need for post-hospitalization rehabilitation, or because of a more permanent decline in health. Physical disability and cognitive impairment are found to be strong predictors of the amount of home care received by older people [16].

### Registries and variables

IPLOS was utilized to identify the study population and retrieve information on the amount of HCS received (in hours per half-year), living situation at the start of each half-year (alone or not alone), and long-term nursing home stays. NorPD provided data on prescriptions for glucose-lowering drugs (GLD) with Anatomical Therapeutic Chemical (ATC) code A10A (insulins and analogues), referred to as insulin, and ATC code A10B (blood glucose-lowering drugs, excl. insulins), referred to as non-insulin GLD. Data from NPR were used to calculate multimorbidity according to the Charlson comorbidity index (CCI), based on discharge diagnoses (using the 10th revision of International Classification of Diseases (ICD-10) codes) from hospitalizations the previous year. The CDR provided information on the time of death (half-year) and the underlying cause of death (as defined by the World Health Organization [17] and stated on the death certificate [18]). In addition, SSB provided age and sex data and merged data from the different registries.

### Definition of diabetes and subgroups

Individuals with ≥ 1 prescription of GLD in the current half-year or the year before were classified as having pharmacologically treated diabetes. Four subgroups were defined within the study population: (1) *not having pharmacologically treated diabetes* (no prescribed GLD current half year or the year before), (2) *non-insulin GLD only* (≥ 1 prescription of non-insulin GLD and no prescription of insulin the current half year or the year before), (3) *both insulin and non-insulin GLD* (≥ 1 prescription with insulin and ≥ 1 prescription of non-insulin GLD during the current half year or the year before) and (4) *insulin only* (≥ 1 prescription with insulin and no prescription of non-insulin GLD current half year or the year before). Each persons’ diabetes status and treatment subgroup were updated every half year from 2009 to 2014.

For language simplicity throughout the paper, the term diabetes labels pharmacologically treated diabetes. Persons diagnosed with diabetes who do not receive pharmacological treatment are included in the group labeled “Not diabetes”.

### Outcomes measures and covariates

Information on the outcome variable, all-cause mortality, was updated 1 st of January and the 1 st of July each year. The underlying cause of death was grouped into eight categories: cancer, CVD, respiratory disease, diabetes, infections, dementia, kidney disease, and other causes. Long-term nursing home stays were treated as a competing event, given that information on mortality was not available for those who moved to nursing homes and because moving to a nursing home was considered to be associated with the risk of death.

The covariate multimorbidity was assessed using the CCI, with Charlson weights updated by Quan et al. [19] and categorized into three groups: 0, 1–2, and >3. CCI scores were updated every half-year. Living situation (alone/not alone) and sex were also treated as covariates.

### Statistical analysis

Data were organized in long format with one observation per person per half-year in the period 2009–2014. Descriptive statistics were used to describe the study population at baseline, which is defined as the first half-year an individual receives HCS during the study period. T-tests, chi-square tests, and median tests were used to compare characteristics between those with and without diabetes, while chi-square tests and linear regression with diabetes subgroups as a categorical independent variable were used to test for differences between diabetes subgroups. All-cause and cause-specific mortality was compared between persons with and without diabetes and between diabetes subgroups using Cox proportional hazards regression with age as the time scale. The category “Not diabetes” was used as the reference group. Each individual was followed from the first half-year with HCS until death or censoring. Individuals were censored if they were alive at the end of follow-up (December 31^st,^ 2014), stopped receiving HCS, or moved permanently to a nursing home. Individuals who restarted use of HCS before the end of follow-up were allowed to re-enter the study population. Regression models for all-cause mortality were stratified by sex and estimated with and without adjustment for multimorbidity (measured by CCI) and living situation (alone/not alone). Furthermore, Cox proportional hazards regression analysis was used to estimate the risk of cause-specific mortality for the three most common underlying causes of death: CVD-death, cancer-death, and respiratory disease-death. Regression models for cause-specific mortality were estimated with and without adjustment for sex and multimorbidity (measured by CCI). For each mortality outcome of interest, individuals who died from other causes, moved to a nursing home, or stopped receiving HCS were censored. Results from Cox regression were reported as hazard ratios (HR) with 95% confidence intervals (CI). In addition to Cox regression, we constructed cumulative incidence curves separately for men and women using the stcompet-command in Stata to take into account competing risk. For all-cause mortality, moving to a nursing home was treated as a competing event, while for cause-specific mortality, both moving to a nursing home and death from other causes were treated as competing events. Bar plots were used to investigate differences in the distribution of causes of death between those with and without diabetes, and between the different subgroups within the study population. All analyses were conducted using STATA version 18. Significance level was defined at *p* < 0.05.

## Results

### Characteristics

The study population comprised 256,400 individuals. Among these, 8.9% were treated with non-insulin GLD only, 2.9% used both insulin and non-insulin GLD, and 2.7% used insulin only. The characteristics of the study population at the start of follow-up are presented in Table 1. Median follow-up time in HCS was 2 years (IQR 1–4) during the study period 2009–2014, and 1.5 years (IQR 1–3) for those who died during this period.

**Table 1:** Characteristics of the study population at start of follow-up (first half-year) (*N* = 256,400)

Variable	Not DM^a^	DM^a^	*p* ^b^	Subgroups of DM^a^			
				Non-insulin GLD^c^ only	Insulin and non-insulin GLD^c^	Insulin only	*p* ^b^
N (% of total population)	219,277 (85.5)	37,123 (14.5)		22,826 (8.9)	7,430 (2.9)	6,867 (2.7)	
Female, n (%)	135,384 (61.7)	19,532 (52.6)	< 0.001	12,418 (54.4)	3,672 (49.4)	3,442 (50.1)	< 0.001
Age, mean (SD)	79.3 (7.0)	77.9 (7.0)	< 0.001	78.6 (6.9)	76.3 (6.8)	77.3 (7.2)	< 0.001
Age group n (%)			< 0.001				< 0.001
65–69	29,291 (13.4)	6,266 (16.9)		3,333 (14.6)	1,578 (21.2)	1,355 (19.7)	
70–74	28,037 (12.8)	5,782 (15.6)		3,187 (14.0)	1,488 (20.0)	1,107 (16.1)	
75–79	41,391 (18.9)	8,053 (21.7)		4,864 (21.3)	1,736 (23.4)	1,453 (21.2)	
80–84	59,617 (27.2)	9,604 (25.9)		6,224 (27.3)	1,697 (22.8)	1,683 (24.5)	
85–90	60,941 (27.8)	7,418 (20.0)		5,218 (22.9)	931 (12.5)	1,269 (18.5)	
Living alone^d^, n (%)	97,266 (56.7)	15,264 (52.0)	< 0.001	9,897 (54.7)	2,689 (46.8)	2,678 (48.3)	< 0.001
Hours of HCS^e^, median (IQR)	13 (3–40)	14 (3–50)	< 0.001	13 (3–40)	17 (4–53)	23 (5–75)	< 0.001
Charlson comorbidity index^d^, n (%)			< 0.001				< 0.001
0	114,312 (82.5)	18,435 (77.8)		11,708 (81.9)	4,033 (74.9)	2,694 (67.2)	
1–2	6,570 (4.7)	1,604 (6.8)		746 (5.2)	419 (7.8)	439 (11.0)	
3–14	17,672 (12.8)	3,643 (15.4)		1,839 (12.9)	930 (17.3)	874 (21.8)	

*All p-values are significant with p-value < 0.05

^a^ *DM* Diabetes mellitus, defined as a person registered in The Norwegian Prescription Database (NorPD) with at least one prescription of Insulins and analogues (A10A) or Blood glucose lowering drugs, excl. insulin (A10B) in the current half year or the year before

^b^ T –tests, chi-square tests and median test are used to investigate the difference between those with and without pharmacologically treated diabetes. Chi-square tests and linear regression with diabetes subgroup as a categorical independent variable are used to test for difference between diabetes subgroups

^c^ *GLD* Glucose lowering drugs

^d^ Variable missing at baseline: Living alone: *n* = 55,489 (21,6%) and Charlson comorbidity index: *n* = 94,164 (36,7%)

^e^*HCS*Home care services

### All cause-mortality risk

**Fig. 1:**
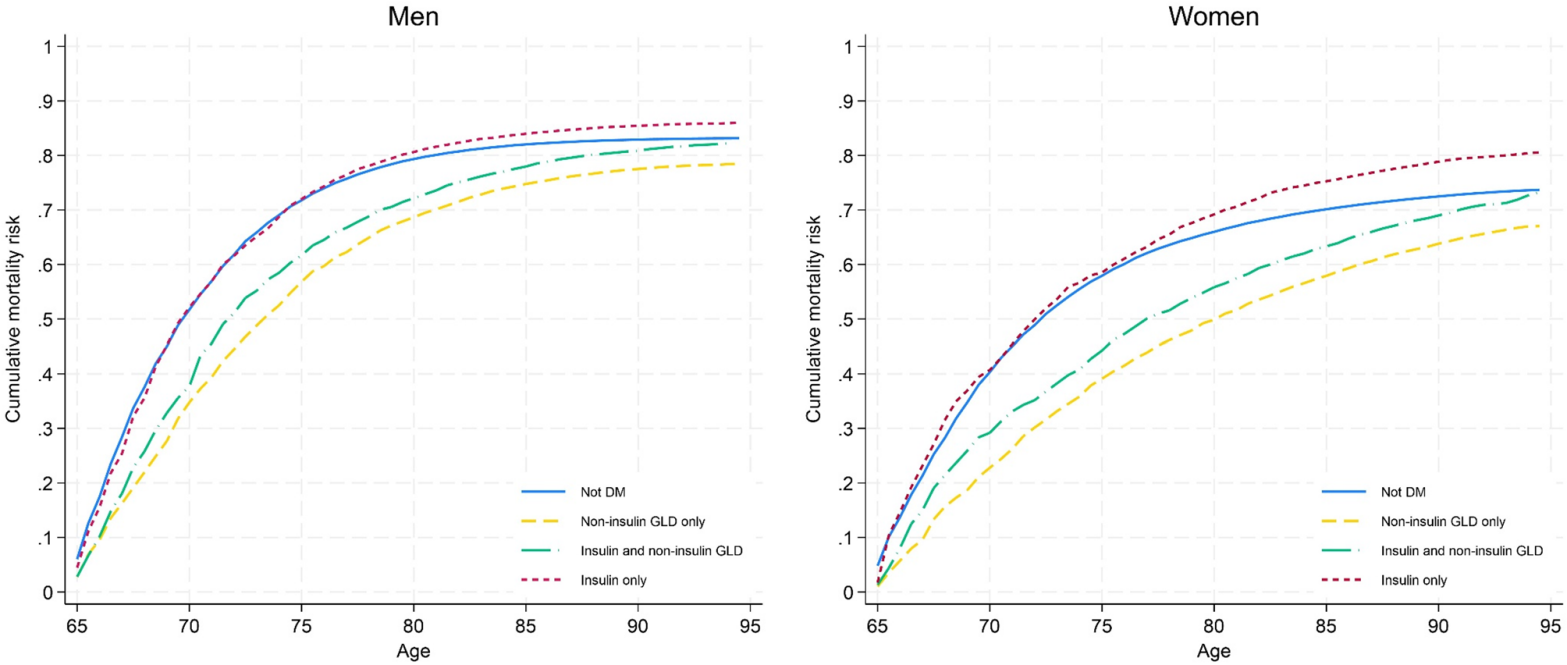
Cumulative risk of all-cause mortality in subgroups with and without diabetes, for men and women. * DM* Diabetes mellitus, defined as a person registered in The Norwegian Prescription Database (NorPD) with at least one prescription of Insulins and analogues (A10A) or Blood glucose lowering drugs, excl. insulin (A10B) in the current half year or the year before. *GLD* Glucose lowering drugs

In total, there were 69,682 individuals who died during the study period. Among men, those with diabetes had a lower all-cause mortality risk compared with men without diabetes (Table 2). When splitting diabetes into sub-groups, the decreased all-cause mortality risk was only present in the “non-insulin GLD only” and “insulin and non-insulin GLD” subgroups. Men using “insulin only” had higher all-cause mortality risk than other men, but the difference was not significant after adjusting for multimorbidity. Overall, all-cause mortality risk in women with diabetes did not significantly differ from that of other women in HCS (Table 2, adjusted models). Women using “insulin only” had a higher all-cause mortality risk compared to women without diabetes, whereas women in the subgroups “non-insulin GLD only” and “insulin and non-insulin GLD” subgroups had a lower all-cause mortality risk. The cumulative all-cause mortality risks for women and men are illustrated in Fig. 1.

**Table 2:** Results of Cox proportional hazards models for all-cause mortality

	Total population	Men			Women		
	Model 1	Model 1	Model 2	Model 3	Model 1	Model 2	Model 3
	HR (CI)	HR (CI)	HR (CI)	HR (CI)	HR (CI)	HR (CI)	HR (CI)
Not DM^a^	1 (Ref)	1 (Ref)	1 (Ref)	1 (Ref)	1 (Ref)	1 (Ref)	1 (Ref)
DM^a^	**1.03 (1.00–1.05)**	**0.92 (0.90–0.95)**	**0.88 (0.85–0.90)**	**0.88 (0.85–0.91)**	**1.05 (1.02–1.09)**	1.00 (0.96–1.03)	1.00 (0.96–1.03)
Subgroups of DM^a^							
Not DM^a^	1 (Ref)	1 (Ref)	1 (Ref)	1 (Ref)	1 (Ref)	1 (Ref)	1 (Ref)
Non-insulin GLD^b^ only	**0.90 (0.87–0.92)**	**0.81 (0.78–0.84)**	**0.83 (0.80–0.86)**	**0.83 (0.80–0.87)**	**0.93 (0.89–0.97)**	**0.94 (0.91–0.98)**	**0.94 (0.90–0.98)**
Insulin and non-insulin GLD^b^	1.03 (0.99–1.07)	**0.93 (0.88–0.98)**	**0.82 (0.78–0.87)**	**0.82 (0.78–0.87)**	1.05 (0.99–1.12)	**0.92 (0.87–0.99)**	**0.93 (0.87–1.00)**
Insulin only	**1.39 (1.34–1.44)**	**1.22 (1.16–1.28)**	1.03 (0.98–1.08)	1.02 (0.97–1.08)	**1.44 (1.37–1.52)**	**1.19 (1.13–1.26)**	**1.18 (1.11–1.25)**

* All models are adjusted for age by using age as the time scale. Model 1: No additional adjustments, Model 2: Adjusted for multimorbidity (measured by categorized Charlson comorbidity index score), Model 3: Adjusted for multimorbidity (measured by categorized Charlson comorbidity index score) and living situation (alone/not alone). Statistically significant (*p*<0.05) hazard ratios highlighted in bold.

^a^ *DM* Diabetes mellitus, defined as a person registered in The Norwegian Prescription Database (NorPD) with at least one prescription of Insulins and analogues (A10A) or Blood glucose lowering drugs, excl. insulin (A10B) in the current half year or the year before

^b^ *GLD* Glucose lowering drugs

### Cause-specific mortality risk and underlying causes of death

**Fig. 2:**
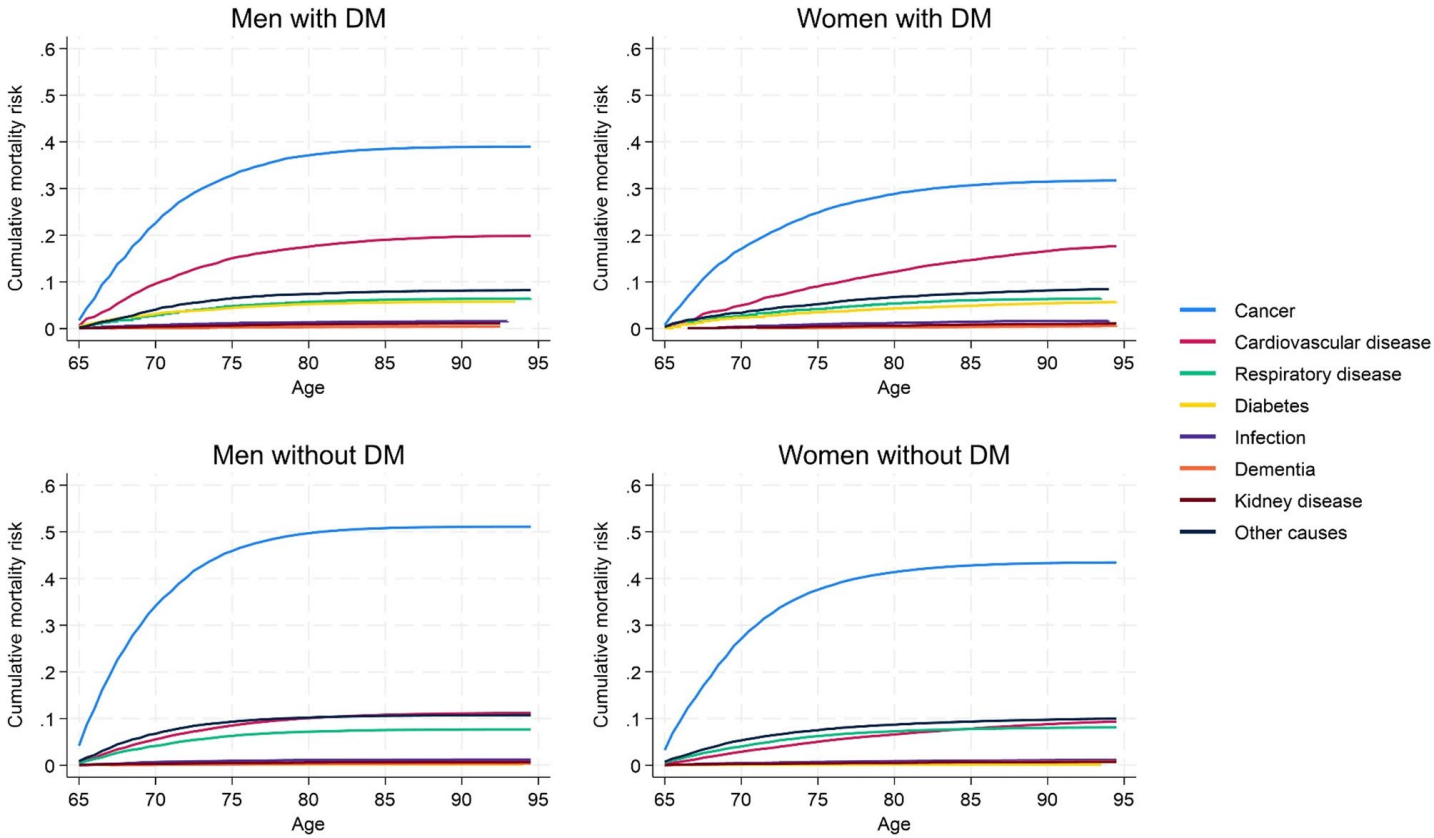
Cumulative risk of cause-specific mortality for men and women with and without diabetes. *DM* Diabetes mellitus, defined as a person registered in The Norwegian Prescription Database (NorPD) with at least one prescription of Insulins and analogues (A10A) or Blood glucose lowering drugs, excl. insulin (A10B) in the current half year or the year before

The cumulative risk of cause-specific mortality for men and women with and without diabetes is presented in Fig. 2. In cause-specific mortality analyses, all the diabetes subgroups had a higher risk of CVD-death compared to persons without diabetes, whereas cancer-related and respiratory disease-related mortality risks were higher among persons without diabetes (Table 3).

**Table 3:** Results of Cox proportional hazards models for Cardiovascular disease-mortality, Cancer-mortality and Respiratory disease-mortality

	CVD^c^-death		Cancer-death		Respiratory disease-death	
	Model 1	Model 2	Model 1	Model 2	Model 1	Model 2
	HR (CI)	HR (CI)	HR (CI)	HR (CI)	HR (CI)	HR (CI)
Not DM^a^	1 (Ref)	1 (Ref)	1 (Ref)	1 (Ref)	1 (Ref)	1 (Ref)
DM^a^	**1.26 (1.21–1.31)**	**1.23 (1.19–1.28)**	**0.73 (0.71–0.76)**	**0.68 (0.66–0.70)**	**0.75 (0.70–0.80)**	**0.67 (0.62–0.72)**
Subgroups of DM^a^						
Not DM^a^	1 (Ref)	1 (Ref)	1 (Ref)	1 (Ref)	1 (Ref)	1 (Ref)
Non-insulin GLD^b^ only	**1.13 (1.08–1.19)**	**1.14 (1.09–1.20)**	**0.63 (0.60–0.66)**	**0.66 (0.63–0.70)**	**0.71 (0.65–0.77)**	**0.71 (0.65–0.77)**
Insulin and non-insulin GLD^b^	**1.23 (1.14–1.33)**	**1.16 (1.07–1.25)**	**0.79 (0.74–0.85)**	**0.68 (0.64–0.73)**	**0.70 (0.61–0.81)**	**0.57 (0.49–0.66)**
Insulin only	**1.66 (1.56–1.77)**	**1.53 (1.43–1.63)**	0.94 (0.89–1.01)	**0.71 (0.67–0.76)**	0.89 (0.79–1.01)	**0.69 (0.60–0.78)**

* All models are adjusted for age by using age as the time scale. Model 1: Adjusted for sex, Model 2: Adjusted for sex and multimorbidity (measured by categorized Charlson comorbidity index score). Statistically significant (*p*<0.05) hazard ratios highlighted in bold.

^a^ *DM * Diabetes mellitus, defined as a person registered in The Norwegian Prescription Database (NorPD) with at least one prescription of Insulins and analogues (A10A) or Blood glucose lowering drugs, excl. insulin (A10B) in the current half year or the year before

^b^ *GLD* Glucose lowering drugs, ^c^ *CVD* Cardiovascular disease

**Fig. 3:**
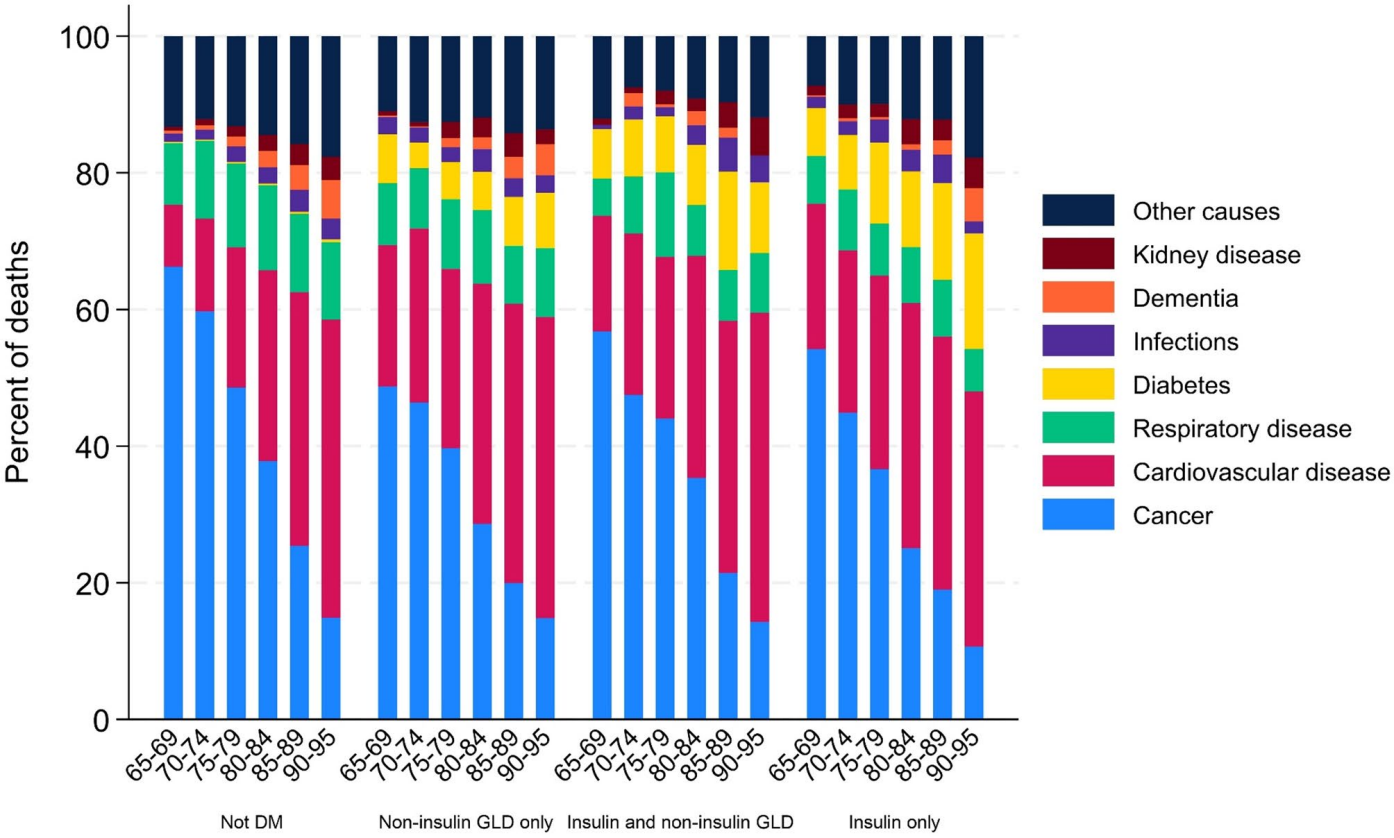
Distribution of underlying cause of death for subgroups with and without diabetes, by age groups. *DM* Diabetes mellitus, defined as a person registered in The Norwegian Prescription Database (NorPD) with at least one prescription of Insulins and analogues (A10A) or Blood glucose lowering drugs, excl. insulin (A10B) in the current half year or the year before. *GLD* Glucose lowering drugs

The three most common underlying causes of death in the HCS population were cancer (38%), CVD (28%), and respiratory disease (11%) (Supplementary Table 1). Figure 3 illustrates the distribution of the underlying cause of death by subgroup and age groups. Cancer was the most common underlying cause of death in the younger age groups, and CVD was most common in the older age groups.

## Discussion

In this nationwide registry study of older people receiving HCS, there were 69,682 deaths during the study period. We found that women using "insulin only" had a higher risk of all-cause mortality than women without diabetes. In contrast, both women and men in the “non-insulin GLD only” and “insulin and non-insulin only” subgroups had lower mortality risks compared to other HCS recipients. Overall, those with diabetes had a higher risk of dying from CVD compared to those without diabetes, while the risk of cancer- and respiratory disease-related mortality was higher among other HCS recipients. The most common underlying causes of death in HCS were cancer, CVD, and respiratory disease.

Sex differences in all-cause mortality risk underscore the particular vulnerability of older women with diabetes. Overall, we found that persons in the “insulin only” subgroup had a higher risk of all-cause mortality compared to other HCS recipients (unadjusted model). However, after adjusting for multimorbidity, the differences in mortality risk narrowed, underlining the important role of comorbid conditions. After stratifying by sex and adjusting for multimorbidity (and living situation), this difference remained significant only among women. The observed sex difference in hazard ratios for “insulin only” relative to persons without diabetes is in line with previous studies showing that the excess mortality risk relative to persons without diabetes is stronger among women [9, 10, 20]. One systematic review and meta-analysis reported that the pooled women-to-men ratio of the standardized mortality ratios for type 1 diabetes was approximately 1.4, indicating 40% greater excess mortality relative to the general population in women compared to men [20]. Another systematic review and meta-analysis that included both type 1 and type 2 diabetes, found (by using women-to-men ratio of RRs) that diabetes was a stronger risk factor in women than men, whereas women had a 13% greater risk of all-cause mortality compared to men with diabetes, along with a 58% excess risk of coronary heart disease [10]. In addition to genetic or biological differences, along with inequalities from cultural and environmental factors, variations in the diagnosis, management, and treatment of diabetes and CVD may contribute to these differences between women and men, according to Wang et al. [10]. The sex differences associated with relative excess vascular risk in women with diabetes are still not fully understood [21, 22]. In the context of older recipients of HCS, one might speculate that behavioral differences or variations in access to healthcare could further contribute to these differences between women and men. Previous studies have identified gender differences within HCS, indicating that women tend to be older, are more likely to live alone, and often depend on children rather than spouses as informal caregivers [23, 24]. Further research is needed to clarify which factors most strongly influence differences in mortality risk between women and men with diabetes receiving HCS.

Women in the “insulin only” subgroup retained an excess all-cause mortality risk compared to other women in HCS, even after adjustments. In our study population, those using “insulin only” were likely to have type 1 diabetes [25], representing 2.7% of the total sample. As life expectancy among those with type 1 diabetes continues to rise, the proportion of older adults within this population is expected to increase [26, 27], thereby expanding the number of people with type 1 diabetes in need of HCS. Previous research indicates that older people with diabetes, both type 1 and type 2 diabetes, are vulnerable to hypoglycemia in the context of HCS [28, 29]. Moreover, severe hypoglycemia among older adults with type 1 diabetes has been associated with impaired cognition [30], and type 1 diabetes has also been associated with an increased risk of dementia [31]. Reduced cognitive function may limit self-management capabilities, making a comprehensive understanding of diabetes management particularly important for healthcare personnel supporting older adults who use insulin [32, 33]. A 32-year follow-up of the Diabetes Control and Complications Trial (DCCT) and the Epidemiology of Diabetes Interventions and Complications (EDIC) study showed that cognitive function declines with age in individuals with type 1 diabetes [34], and this decline is further influenced by glycemic control and blood pressure. Probably, the group using “insulin only” also includes people with type 2 diabetes with complications like renal failure that can contraindicate the use of several non-insulin GLD. The increased mortality risk observed in older women using “insulin only” suggests that this subgroup is particularly vulnerable within HCS. We believe that the observed differences in mortality risk between diabetes subgroups underscore the need for enhanced expertise in diabetes screening, management, and prevention, alongside the provision of person-centered care, in line with standards for all older adults with complex needs. These findings may inform more targeted interventions at the system, institutional, and individual levels. Such interventions should include efficient care pathways to ensure timely follow-up and treatment adjustments in accordance with diabetes management guidelines for primary healthcare, incorporate lifelong learning for healthcare personnel, and employ shared decision-making for active patient and caregiver engagement.

Interestingly, persons with diabetes using “non-insulin GLD only” and “insulin and non-insulin GLD” had lower all-cause mortality risks in both women and men compared to other HCS recipients. These findings were unexpected, as diabetes is generally associated with an increased mortality risk in studies comparing individuals with and without diabetes [9]. One possible explanation is that other HCS recipients may have other severe or complex conditions, e.g. cancer, contributing to an increased mortality risk. We previously found that individuals with diabetes treated with “non-insulin GLD only” and “insulin and non-insulin GLD” had a lower risk of long-term nursing home stays compared to other recipients of HCS [35], which aligns with this explanation. Still, the observed difference in all-cause mortality risk between the various diabetes treatment groups underscores the importance of providing individualized health care to older people in HCS. Diabetes is a highly heterogeneous condition, and screening for geriatric syndromes is recommended [26]. The subgroups using non-insulin GLD are likely to consist of persons with type 2 diabetes [26]. Strain et al. [36] also recommend that the assessment of frailty in older people with type 2 diabetes to be performed regularly and considered when making therapeutic choices and setting glycemic targets. Such assessments could enhance the delivery of person-centered care.

Persons with diabetes in all treatment groups had a higher risk of death due to CVD, compared to those without diabetes. Although landmark trials (e.g. the United Kingdom prospective diabetes study (UKPDS) and the Diabetes Control and Complications Trial (DCCT)) indicate that good glucose control may protect against cardiovascular outcomes [37] and that a “legacy effect” of glycemic control may be observed long after the trials concluded, an increased CVD-mortality is expected. However, data from recent years indicate a larger decrease in CVD-mortality than in cancer mortality among patients with diabetes in the general population [6]. In an epidemiologic analysis of linked primary care records, Pearson-Stuttard et al. [8] found that in the period 2001 to 2018, vascular death rates (rate per 1000 person years) declined among both people with and without diabetes, and cancer was the most common cause of death also among persons with diabetes in 2018. Diabetes is associated with several types of cancer, in particular liver, pancreas, endometrium, colon, breast, and bladder cancers [38]. Furthermore, others have found that compared to those without diabetes, individuals with diabetes have an increased risk of death not only from CVD but also from cancer, respiratory diseases, and other causes [39]. This is in contrast with our findings, where the risk of death due to cancer and respiratory disease was lower among those with diabetes. Thus, this underlines the differences between the general population and an HCS population, as the comparison group in our study consists of other HCS recipients. Unlike the general population, the HCS population is composed of older people with various levels of care needs, many of whom also experience multimorbidity. For many without diabetes, chronic obstructive lung disease or cancer - often accompanied by palliative care related to a cancer diagnosis, may represent the primary reason for receiving HCS. Consequently, when comparing those with diabetes to other HCS recipients, it is not unexpected that mortality risk from respiratory disease and cancer is not increased.

The most common underlying causes of death in this HCS population were cancer (38%), CVD (28%), and respiratory disease (11%). In a previous study with data from the general Norwegian population, the most common underlying causes of death in the period 2009–2013 were found to be diseases of the circulatory system (31%), cancer (26%), and respiratory diseases (10%) [40]. We found among those with diabetes that the proportion of people having CVD and cancer as an underlying cause of death was equal (32%). However, this varied by age, and CVD predominated as the underlying cause of death in the older age group, while cancer predominated as the underlying cause of death in the younger age group of our study population (< 85 years). In Norway, there has been inadequate reporting of diagnoses among recipients of HCS to the primary health care registries [41, 42], and subsequently lack of information on the prevalence of different diseases. However, in a recent study, the prevalence of cancer was estimated to be 21.3% among recipients of home-based services [42]. Thus, as cancer is relatively common among HCS recipients, increased cancer mortality is expected in this population compared to the general population. Our findings contribute new information on the distribution of underlying causes of death in different diabetes subgroups in HCS.

### Strengths and limitations

By using nationwide registry data, our study benefits from comprehensive data encompassing all individuals 65 years or older who receive HCS in Norway, which represents a major strength. The use of registry data and ATC codes to identify individuals with pharmacologically treated diabetes is also a strength of this study. However, missing information on diabetes diagnosis in IPLOS and potentially in HCS records limited our ability to include persons with diabetes not using pharmacological treatment, as well as to distinguish between type 1 diabetes and type 2 diabetes diagnosis. Such information could have contributed to a clearer understanding of the observed differences between subgroups. Especially in the subgroup “insulin only”, there might be both individuals with type 1 diabetes and individuals with type 2 diabetes with, for example, kidney failure, preventing them from using non-insulin GLD. Still, we believe that the use of information on prescriptions to identify the different treatment subgroups contributes important information to the understanding of diabetes among older people in HCS. Moreover, the CCI was utilized as a measurement of the covariate multimorbidity, and there are some limitations related to this. As the CCI is based on diagnoses reported during hospital stays, only the most severe multimorbidity that required hospitalization is included. In addition, “diabetes with complications” is included in the CCI and might contribute to a higher level of multimorbidity for those with diabetes. However, this is similar for the comparison group, other HCS recipients, with other diagnoses included in the CCI, such as cancer, dementia, and chronic pulmonary disease [19]. Another limitation is the lack of information on body mass index, smoking history and alcohol consumption, which could be important confounders of the relationship of diabetes with all-cause and cause-specific mortality risk. However, as the primary aim of this study was to describe differences between people with and without diabetes, rather than to investigate deeper causal relationships, we consider this limitation to be less important. Another limitation is the large number of statistical tests conducted, which may result in findings attributed to chance due to multiple testing, increasing the risk of type 1 errors. Furthermore, it is a limitation that we do not have data from the years after 2014. There has been an increase in different treatment options for people with type 2 diabetes in recent years [36]. Although medical therapies have advanced since these data were collected, we believe these treatment improvements are unlikely to have altered the all-cause or cause-specific mortality patterns observed in this nationwide registry study.

## Conclusion

In this nationwide registry study of older people receiving HCS, we observed differences in mortality risk based on diabetes status and treatment regimens. Women prescribed insulin only showed a higher all-cause mortality risk compared to women without diabetes. Sex differences in mortality risk underscore the vulnerability of older women with diabetes. After adjusting for multimorbidity, the differences in mortality risk between persons treated with "insulin only" and those without diabetes narrowed, underlining the important role of comorbid conditions in outcomes. Cancer predominated as the underlying cause of death in individuals < 85 years of age at time of death, although persons with diabetes had an increased risk of CVD-death compared to those without diabetes. Insulin-only users appeared especially vulnerable due to the risk of all-cause mortality, emphasizing the need for tailored follow-up, coordinated care and careful monitoring. Our research contributes to a clearer understanding of mortality risk in patients with diabetes receiving HCS and may facilitate more targeted interventions.

## Supplementary Information

Below is the link to the electronic supplementary material.

Supplementary Material 1

## Data Availability

The datasets generated and/or analysed during the current study are not publicly available due to data protection regulations. Personal data protection legislation and the approval from the Regional Ethical Committee prohibits data sharing for the purpose of reproducing the results. Researchers can apply for ethical approval and obtain data from the registry holders (service@helsedata.no).

## Abbreviations

ATC: Anatomical Therapeutic Chemical
CCI: Charlson Comorbidity Index
CDR: Cause of death registry
CI: Confidence interval
CVD: Cardiovascular disease
DM: Diabetes mellitus
GLD: Glucose-lowering drugs
HCS: Home care services
HR: Hazard ratios
ICD-10: The 10th revision of International Classification of Diseases codes
IPLOS: The Norwegian Information System for the Nursing and Care Sector
NorPD: The Norwegian Prescription Database
NPR: Norwegian Patient Registry
SSB: Statistics Norway

## References

[1] Prince MJ, Wu F, Guo Y, Robledo LMG, O’Donnell M, Sullivan R, et al. The burden of disease in older people and implications for health policy and practice. Lancet. 2015;385(9967):549–62. 10.1016/S0140-6736(14)61347-7. 25468153 10.1016/S0140-6736(14)61347-7

[2] Carrasco-Ribelles LA, Roso-Llorach A, Cabrera-Bean M, Costa-Garrido A, Zabaleta-del-Olmo E, Toran-Monserrat P, et al. Dynamics of Multimorbidity and frailty, and their contribution to mortality, nursing home and home care need: A primary care cohort of 1 456 052 ageing people. Eclinicalmedicine. 2022;52:101610. 10.1016/j.eclinm.2022.101610. 36034409 10.1016/j.eclinm.2022.101610 PMC9399153

[3] Sinclair A, Saeedi P, Kaundal A, Karuranga S, Malanda B, Williams R. Diabetes and global ageing among 65–99-year-old adults: findings from the international diabetes federation diabetes atlas. Diabetes Res Clin Pract. 2020;162:108078. 10.1016/j.diabres.2020.108078. 32068097 10.1016/j.diabres.2020.108078

[4] Baena-Díez JM, Peñafiel J, Subirana I, Ramos R, Elosua R, Marín-Ibañez A, et al. Risk of cause-specific death in individuals with diabetes: a competing risks analysis. Diabetes Care. 2016;39(11):1987–95. 10.2337/dc16-0614. 27493134 10.2337/dc16-0614

[5] Ali MK, Pearson-Stuttard J, Selvin E, Gregg EW. Interpreting global trends in type 2 diabetes complications and mortality. Diabetologia. 2022;65(1):3–13. 10.1007/s00125-021-05585-2. 34837505 10.1007/s00125-021-05585-2 PMC8660730

[6] Gregg EW, Cheng YJ, Srinivasan M, Lin J, Geiss LS, Albright AL, et al. Trends in cause-specific mortality among adults with and without diagnosed diabetes in the USA: an epidemiological analysis of linked National survey and vital statistics data. Lancet. 2018;391(10138):2430–40. 10.1016/S0140-6736(18)30314-3. 29784146 10.1016/S0140-6736(18)30314-3

[7] Gregg EW, Pratt A, Owens A, Barron E, Dunbar-Rees R, Slade ET, et al. The burden of diabetes-associated multiple long-term conditions on years of life spent and lost. Nat Med. 2024;30(10):2830–7. 10.1038/s41591-024-03123-2. 39090411 10.1038/s41591-024-03123-2 PMC11485235

[8] Pearson-Stuttard J, Bennett J, Cheng YJ, Vamos EP, Cross AJ, Ezzati M, et al. Trends in predominant causes of death in individuals with and without diabetes in England from 2001 to 2018: an epidemiological analysis of linked primary care records. Lancet Diabetes Endocrinol. 2021;9(3):165–73. 10.1016/S2213-8587(20)30431-9. 33549162 10.1016/S2213-8587(20)30431-9 PMC7886654

[9] Forbes A. Reducing the burden of mortality in older people with diabetes: a review of current research. Front Endocrinol. 2020;11:133. 10.3389/fendo.2020.00133. 10.3389/fendo.2020.00133 PMC7089919 32256448

[10] Wang Y, O’Neil A, Jiao Y, Wang L, Huang J, Lan Y, et al. Sex differences in the association between diabetes and risk of cardiovascular disease, cancer, and all-cause and cause-specific mortality: a systematic review and meta-analysis of 5,162,654 participants. BMC Med. 2019;17:1–18. 10.1186/s12916-019-1355-0. 31296205 10.1186/s12916-019-1355-0 PMC6625042

[11] Forbes A, Murrells T, Sinclair A. Examining factors associated with excess mortality in older people (age ≥ 70 years) with diabetes–a 10-year cohort study of older people with and without diabetes. Diabet Med. 2017;34(3):387–95. 10.1111/dme.13132. 27087619 10.1111/dme.13132

[12] Genet N, Boerma WG, Kringos DS, Bouman A, Francke AL, Fagerström C, et al. Home care in europe: a systematic literature review. BMC Health Serv Res. 2011;11:1–14. 10.1186/1472-6963-11-207. 21878111 10.1186/1472-6963-11-207 PMC3170599

[13] Næss G, Kirkevold M, Hammer W, Straand J, Wyller TB. Nursing care needs and services utilised by home-dwelling elderly with complex health problems: observational study. BMC Health Serv Res. 2017;17:1–10. 10.1186/s12913-017-2600-x. 28899369 10.1186/s12913-017-2600-x PMC5596938

[14] Kristinsdóttir IV, Jónsson PV, Hjaltadóttir I, Bjornsdottir K. Changes in home care clients’ characteristics and home care in five European countries from 2001 to 2014: comparison based on InterRAI-Home care data. BMC Health Serv Res. 2021;21(1):1–12. 10.1186/s12913-021-07197-3. 34715850 10.1186/s12913-021-07197-3 PMC8555210

[15] Holm SG, Mathisen TA, Sæterstrand TM, Brinchmann BS. Allocation of home care services by municipalities in norway: a document analysis. BMC Health Serv Res. 2017;17(1):1–10. 10.1186/s12913-017-2623-3. 28938892 10.1186/s12913-017-2623-3 PMC5610450

[16] Døhl Ø, Garåsen H, Kalseth J, Magnussen J. Factors associated with the amount of public home care received by elderly and intellectually disabled individuals in a large Norwegian municipality. Health Soc Care Commun. 2016;24(3):297–308. 10.1111/hsc.12209. 10.1111/hsc.12209 25706800

[17] World Health Organization. Classification of diseases and cause of death. World Health Organization. https://www.who.int/standards/classifications/classification-of-diseases/cause-of-death Accessed 13.06.25.

[18] Pedersen AG, Ellingsen CL. Data quality in the Causes of Death Registry. Tidsskrift for Den norske legeforening. 2015. 10.4045/tidsskr.14.1065 10.4045/tidsskr.14.1065 25947599

[19] Quan H, Li B, Couris CM, Fushimi K, Graham P, Hider P, et al. Updating and validating the Charlson comorbidity index and score for risk adjustment in hospital discharge abstracts using data from 6 countries. Am J Epidemiol. 2011;173(6):676–82. 10.1093/aje/kwq433. 21330339 10.1093/aje/kwq433

[20] Huxley RR, Peters SA, Mishra GD, Woodward M. Risk of all-cause mortality and vascular events in women versus men with type 1 diabetes: a systematic review and meta-analysis. Lancet Diabetes Endocrinol. 2015;3(3):198–206. 10.1016/S2213-8587(14)70248-7. 25660575 10.1016/S2213-8587(14)70248-7

[21] Roche MM, Wang PP. Sex differences in all-cause and cardiovascular mortality, hospitalization for individuals with and without diabetes, and patients with diabetes diagnosed early and late. Diabetes Care. 2013;36(9):2582–90. 10.2337/dc12-1272. 23564923 10.2337/dc12-1272 PMC3747934

[22] Gnatiuc L, Herrington WG, Halsey J, Tuomilehto J, Fang X, Kim HC, et al. Sex-specific relevance of diabetes to occlusive vascular and other mortality: a collaborative meta-analysis of individual data from 980 793 adults from 68 prospective studies. Lancet Diabetes Endocrinol. 2018;6(7):538–46. 10.1016/S2213-8587(18)30079-2. 29752194 10.1016/S2213-8587(18)30079-2 PMC6008496

[23] Gruneir A, Forrester J, Camacho X, Gill SS, Bronskill SE. Gender differences in home care clients and admission to long-term care in Ontario, canada: a population-based retrospective cohort study. BMC Geriatr. 2013;13(1):48. 10.1186/1471-2318-13-48. 23678949 10.1186/1471-2318-13-48 PMC3679828

[24] Dorin L, Krupa E, Metzing S, Büscher A. Gender disparities in German home-care arrangements. Scand J Caring Sci. 2016;30(1):164–74. 10.1111/scs.12236. 26036651 10.1111/scs.12236

[25] Strøm H, Selmer R, Birkeland KI, Schirmer H, Berg TJ, Jenum AK, et al. No increase in new users of blood glucose-lowering drugs in Norway 2006–2011: a nationwide prescription database study. BMC Public Health. 2014;14(1):1–9. 10.1186/1471-2458-14-520. 24886413 10.1186/1471-2458-14-520 PMC4045953

[26] American Diabetes Association Professional Practice Committee. 13. Older Adults: Standards of Care in Diabetes—2025. Diabetes Care. 2024;48(Supplement_1):S266–S82. 10.2337/dc25-S013 10.2337/dc25-S013 PMC11635042 39651977

[27] Yang K, Yang X, Jin C, Ding S, Liu T, Ma B, et al. Global burden of type 1 diabetes in adults aged 65 years and older, 1990–2019: population based study. BMJ. 2024. 10.1136/bmj-2023-078432. 385. 38866425 10.1136/bmj-2023-078432 PMC11167563

[28] Fløde M, Hermann M, Haugstvedt A, Søfteland E, Igland J, Åsberg A, et al. High number of hypoglycaemic episodes identified by CGM among home-dwelling older people with diabetes: an observational study in Norway. BMC Endocr Disorders. 2023;23(1):218. 10.1186/s12902-023-01472-6. 10.1186/s12902-023-01472-6 PMC10566065 37817166

[29] Hermann M, Heimro LS, Haugstvedt A, Hernar I, Sigurdardottir AK, Graue M. Hypoglycaemia in older home-dwelling people with diabetes-a scoping review. BMC Geriatr. 2021;21(1):1–11. 10.1186/s12877-020-01961-6. 33413148 10.1186/s12877-020-01961-6 PMC7792330

[30] Lacy ME, Gilsanz P, Eng C, Beeri MS, Karter AJ, Whitmer RA. Severe hypoglycemia and cognitive function in older adults with type 1 diabetes: the study of longevity in diabetes (SOLID). Diabetes Care. 2020;43(3):541–8. 10.2337/dc19-0906. 31882410 10.2337/dc19-0906 PMC7035586

[31] Li L, Wong D, Fisher CA, Conn JJ, Wraight PR, Davies A, et al. Increased risk of dementia in type 1 diabetes: A systematic review with meta-analysis. Diabetes Res Clin Pract. 2025;112043. 10.1016/j.diabres.2025.112043. 10.1016/j.diabres.2025.112043 39965720

[32] Munshi MN, Florez H, Huang ES, Kalyani RR, Mupanomunda M, Pandya N, et al. Management of diabetes in Long-term care and skilled nursing facilities: A position statement of the American diabetes association. Diabetes Care. 2016;39(2):308–18. 10.2337/dc15-2512. 26798150 10.2337/dc15-2512 PMC5317234

[33] Haltbakk J, Graue M, Harris J, Kirkevold M, Dunning T, Sigurdardottir AK. Integrative review: patient safety among older people with diabetes in home care services. J Adv Nurs. 2019;75(11):2449–60. 10.1111/jan.13993. 30835874 10.1111/jan.13993

[34] Jacobson AM, Ryan CM, Braffett BH, Gubitosi-Klug RA, Lorenzi GM, Luchsinger JA, et al. Cognitive performance declines in older adults with type 1 diabetes: results from 32 years of follow-up in the DCCT and EDIC study. Lancet Diabetes Endocrinol. 2021;9(7):436–45. 10.1016/S2213-8587(21)00086-3. 34051936 10.1016/S2213-8587(21)00086-3 PMC8583716

[35] Teigland T, Igland J, Graue M, Blytt KM, Haltbakk J, Tell GS, et al. Associations between diabetes and risk of short-term and long-term nursing home stays among older people receiving home care services: A nationwide registry study. BMC Geriatr. 2024;24(1):814. 10.1186/s12877-024-05403-5. 39385069 10.1186/s12877-024-05403-5 PMC11462714

[36] Strain WD, Down S, Brown P, Puttanna A, Sinclair A. Diabetes and frailty: an expert consensus statement on the management of older adults with type 2 diabetes. Diabetes Therapy. 2021;12(5):1227–47. 10.1007/s13300-021-01035-9. 33830409 10.1007/s13300-021-01035-9 PMC8099963

[37] Murray P, Chune GW, Raghavan VA. Legacy effects from DCCT and UKPDS: what they mean and implications for future diabetes trials. Curr Atheroscler Rep. 2010;12(6):432–9. 10.1007/s11883-010-0128-1. 20652839 10.1007/s11883-010-0128-1

[38] Giovannucci E, Harlan DM, Archer MC, Bergenstal RM, Gapstur SM, Habel LA, et al. Diabetes and cancer: a consensus report. Cancer J Clin. 2010;60(4):207–21. 10.3322/caac.20078. 10.3322/caac.20078 20554718

[39] Gordon-Dseagu VL, Shelton N, Mindell J. Diabetes mellitus and mortality from all-causes, cancer, cardiovascular and respiratory disease: evidence from the health survey for England and Scottish health survey cohorts. J Diabetes Complicat. 2014;28(6):791–7. 10.1016/j.jdiacomp.2014.06.016. 10.1016/j.jdiacomp.2014.06.016 25104237

[40] Michel YA, Aas E, Augestad LA, Burger E, Thoresen L, Bjørnelv GMW. Healthcare use and costs in the last six months of life by level of care and cause of death. BMC Health Serv Res. 2024;24(1):688. 10.1186/s12913-024-10877-5. 38816869 10.1186/s12913-024-10877-5 PMC11140868

[41] Beyrer S, Otnes B, Karlsen HT, Kvalitet i IPLOS-registeret. 2017. Gjennomgang av datakvaliteten på kommunenes IPLOS-rapportering. 2018. doi: https://core.ac.uk/download/pdf/249953966.pdf

[42] Bakken IJ, Heyeraas TN, Døhl Ø, Rostoft S, Ariansen I, Burrell LV. Diagnoses among long-term care recipients in norway: a nation-wide registry study on prevalence and data completeness. Scand J Public Health. 2025;14034948251339873. 10.1177/14034948251339873. 10.1177/14034948251339873 PMC13323933 40396564

