# Correction: All-cause and cause-specific mortality in older people with and without diabetes in Norwegian home care services: a nationwide registry study

**DOI:** 10.1186/s12877-026-07053-1

**Published:** 2026-02-12

**Authors:** Tonje Teigland, Jannicke Igland, Kjersti M. Blytt, Johannes Haltbakk, Kåre I. Birkeland, Truls Østbye, Marit Kirkevold, Marjolein M. Iversen

**Affiliations:** 1 Department of Health and Caring Sciences, Western Norway University of Applied Sciences, Bergen, Norway; 2 Department of Global Public Health and Primary Care, University of Bergen, Bergen, Norway; 3 Institute of Clinical Medicine, University of Oslo, Oslo, Norway; 4 Department of Transplantation Medicine, Oslo University Hospital, Oslo, Norway; 5 Department of Family Medicine and Community Health, Duke University, Durham, NC USA; 6 Faculty of Health Sciences, Oslo Metropolitan University, Oslo, Norway

**Correction** **:** **BMC Geriatr 25, 999 (2025)**

10.1186/s12877-025-06685-z

Following publication of the original article [1], the authors reported the below errors in the main text.

1. The 'Statistical Analysis' section was mistakenly placed at the beginning of the Methods. It should be moved to the end, after the 'Outcome Measures and Covariates' section.
2. In Table 2 several confidence intervals are displayed on a separate line below the corresponding estimates, rather than on the same line.

**Table 2:** Results of Cox proportional hazards models for all-cause mortality

	**Total population**	**Men**			**Women**		
	**Model 1**	**Model 1**	**Model 2**	**Model 3**	**Model 1**	**Model 2**	**Model 3**
	**HR (CI)**	**HR (CI)**	**HR (CI)**	**HR (CI)**	**HR (CI)**	**HR (CI)**	**HR (CI)**
Not DM^a^	1 (Ref)	1 (Ref)	1 (Ref)	1 (Ref)	1 (Ref)	1 (Ref)	1 (Ref)
DM^a^	**1.03 (1.00–1.05)**	**0.92 (0.90–0.95)**	**0.88 (0.85–0.90)**	**0.88 (0.85–0.91)**	**1.05 (1.02–1.09)**	1.00 (0.96–1.03)	1.00 (0.96–1.03)
Subgroups of DM^a^							
Not DM^a^	1 (Ref)	1 (Ref)	1 (Ref)	1 (Ref)	1 (Ref)	1 (Ref)	1 (Ref)
Non-insulin GLD^b^ only	**0.90 (0.87–0.92)**	**0.81 (0.78–0.84)**	**0.83 (0.80–0.86)**	**0.83 (0.80–0.87)**	**0.93 (0.89–0.97)**	**0.94 (0.91–0.98)**	**0.94 (0.90–0.98)**
Insulin and non-insulin GLD^b^	1.03 (0.99–1.07)	**0.93 (0.88–0.98)**	**0.82 (0.78–0.87)**	**0.82 (0.78–0.87)**	1.05 (0.99–1.12)	**0.92 (0.87–0.99)**	**0.93 (0.87–1.00)**
Insulin only	**1.39 (1.34–1.44)**	**1.22 (1.16–1.28)**	1.03 (0.98–1.08)	1.02 (0.97–1.08)	**1.44 (1.37–1.52)**	**1.19 (1.13–1.26)**	**1.18 (1.11–1.25)**

*All models are adjusted for age by using age as the time scale. Model 1: No additional adjustments, Model 2: Adjusted for multimorbidity (measured by categorized Charlson comorbidity index score), Model 3: Adjusted for multimorbidity (measured by categorized Charlson comorbidity index score) and living situation (alone/not alone). Statistically significant (*p* < 0.05) hazard ratios highlighted in bold

^a^*DM* Diabetes mellitus, defined as a person registered in The Norwegian Prescription Database (NorPD) with at least one prescription of Insulins and analogues (A10A) or Blood glucose lowering drugs, excl. insulin (A10B) in the current half year or the year before

^b^*GLD* Glucose lowering drugs

The original article has been corrected.

## References

[1] Teigland T, Igland J, Blytt KM, et al. All-cause and cause-specific mortality in older people with and without diabetes in Norwegian home care services: a nationwide registry study. BMC Geriatr. 2025;25:999. 10.1186/s12877-025-06685-z. 41350985 10.1186/s12877-025-06685-z PMC12681130

