# Supplementary material for: All-cause and cause-specific mortality in older people with and without diabetes in Norwegian home care services: a nationwide registry study
Source: BMC Geriatr. 2025 Dec 5;25:999. doi: 10.1186/s12877-025-06685-z (PMC12681130; doi:10.1186/s12877-025-06685-z)
Supplement: Supplementary file 1 — Supplementary Material 1 [file 12877_2025_6685_MOESM1_ESM.docx]

**Additional file 1**

**Supplementary Table 1: Distribution of underlying causes of death for subgroups with and without diabetes**

| Variable | **Total population** | **Not DM^a^** | **DM^a^** | **Subgroups of DM^a^** | | |
| --- | --- | --- | --- | --- | --- | --- |
|  |  |  |  | **Non-insulin GLD^b^ only** | **Insulin and non-insulin GLD^b^** | **Insulin only** |
| N of deaths | 69,682 | 58,382 | 11,300 | 5,808 | 2,345 | 3,147 |
| Cancer, n (%) | **26,310 (37.8)** | **22,669 (38.8)** | **3,641 (32.2)** | 1,760 (30.3) | **889 (37.9)** | **992 (31.5)** |
| Cardiovascular disease, n (%) | 19,724 (28.3) | 16,099 (27.6) | 3,625 (32.1) | **1,968 (33.9)** | 676 (28.8) | 981 (31.2) |
| Respiratory disease, n (%) | 7,744 (11.1) | 6,743 (11.6) | 1,001 (8.9) | 557 (9.6) | 195 (8.3) | 249 (7.9) |
| Diabetes, n (%) | 1,100 (1.6) | 158 (0.3) | 942 (8.3) | 359 (6.2) | 226 (9.6) | 357 (11.3) |
| Infections, n (%) | 1,742 (2.5) | 1,431 (2.5) | 311 (2.8) | 157 (2.7) | 61 (2.6) | 93 (3.0) |
| Dementia, n (%) | 1,664 (2.4) | 1,482 (2.5) | 182 (1.6) | 117 (2.0) | 28 (1.2) | 37 (1.2) |
| Kidney, n (%) | 1,561 (2.2) | 1,280 (2.2) | 281 (2.5) | 144 (2.5) | 51 (2.2) | 86 (2.7) |
| Other, n (%) | 9,837 (14.1) | 8,520 (14.6) | 1,317 (11.7) | 746 (12.8) | 219 (9.3) | 352 (11.2) |

^a^ DM = Diabetes mellitus, defined as a person registered in The Norwegian Prescription Database (NorPD) with at least one prescription of Insulins and analogues (A10A) or Blood glucose lowering drugs, excl. insulin (A10B) in the current half year or the year before. ^b^ GLD = Glucose lowering drugs.
